# Single-center experience with the ClotTriever BOLD catheter for deep vein thrombosis percutaneous mechanical thrombectomy of the lower extremity

**DOI:** 10.3389/fsurg.2023.1268338

**Published:** 2023-11-02

**Authors:** Lorena P. De Marco Garcia

**Affiliations:** ^1^ Division of Vascular Surgery, Department of Surgery, Plainview Hospital, Northwell Health System, Plainview, NY, United States; ^2^ Department of Surgery, Donald and Barbara Zucker School of Medicine at Hofstra/Northwell, Hempstead, NY, United States

**Keywords:** deep vein thrombosis, lower extremity, percutaneous mechanical thrombectomy, chronic thrombus, ClotTriever BOLD catheter

## Abstract

**Background:**

The ClotTriever System is a percutaneous mechanical thrombectomy system used to treat deep vein thrombosis (DVT). The BOLD catheter is a newer compatible component with a modified coring element for which reported outcomes are limited. This retrospective study aims to assess the preliminary procedural safety and success data for patients treated with the BOLD catheter.

**Methods:**

All consecutive patients with symptomatic lower extremity DVT who underwent thrombectomy with the BOLD catheter between 23 November 2021 and 26 June 2022 at a single center were included. Baseline and procedural characteristics were reported. The primary outcome, intraprocedural safety, was assessed by a chart review of recorded intraprocedural adverse events (AEs) or device malfunction. The secondary outcome, procedural success, was defined as ≥75% reduction in the total occlusion across treated venous segments. This was assessed by an interventionalist review of pre- and postprocedural venograms. Additional outcomes included length of postprocedural hospital stay and assessment of AEs at discharge and a 30-day follow-up visit.

**Results:**

Eleven patient cases were reviewed. The median patient age was 65 years, the majority were women, and all were treated unilaterally. All procedures were completed in a single session without intraprocedural AEs or device malfunction. The median procedural blood loss was 50 ml. A review of pre- and postprocedural venograms showed that 35 venous segments were treated, including the femoral (*n* = 9), common femoral (*n* = 9), external iliac (*n* = 10), and common iliac (*n* = 7) veins. Procedural success was achieved in 10 patients (90.9%), and the median reduction rate in venous occlusion was 100%. The median length of postprocedural hospital stay was 1 day, and no AEs were noted at discharge (*N* = 11). One adverse event occurred among the eight patients who completed their follow-up visit. A patient with advanced-stage cancer and medication failure had a recurrent DVT 13 days postprocedure, which was not related to the device or procedure.

**Conclusions:**

No safety concerns concerning the BOLD catheter were raised during the review of the cases included in this analysis, and the device was successful in reducing venous occlusion in patients with symptomatic proximal lower extremity DVT.

## 1. Introduction

Venous thromboembolism (VTE) affects approximately 900,000 people in the United States annually, corresponding to 100,000 deaths and up to $10 billion in healthcare costs (1). VTE includes pulmonary embolism (PE) and deep vein thrombosis (DVT), a blood clot that forms in the deep veins of the limbs and commonly affects the lower extremities. The burden of DVT-associated morbidity is high, with approximately one in three patients experiencing long-term complications (2). Collectively referred to as post-thrombotic syndrome (PTS), long-term symptoms following acute DVT include swelling, discoloration, and pain in the affected limb. Within 2 years of DVT, PTS significantly increases healthcare costs, and the rate of occurrence has been reported to be as high as 60% (3, 4). PTS is a chronic condition with limited treatment options and is independently associated with a diminished quality of life (5). In addition, PTS is the most common cause of the development of venous leg ulcers, affecting up to 10% of patients within 2 years (3).

Following DVT, residual venous obstruction is common and associated with an increased risk of poor outcomes, including death, recurrent VTE, and greater severity PTS (6–8). Residual obstruction develops when the DVT thrombus does not fully resolve. Interventional DVT treatment aimed at removing thrombus may improve the longer-term outcomes of patients by reducing venous obstruction (9, 10). The ClotTriever System (Inari Medical, Irvine, CA) is a percutaneous mechanical thrombectomy (PMT) device shown to be effective in removing thrombus in veins affected by DVT with acute or chronic presentation (11–14). The ClotTriever BOLD catheter (Inari Medical, Irvine, CA, USA) is a newer addition to the system that has a coring element with an increased wall thickness compared with the legacy device. However, the procedural steps do not differ from those for the legacy device (15). Case reports using the BOLD catheter have been published (16, 17). However, a larger population is needed to evaluate the design performance regarding acute safety and success. This study aimed to assess the procedural outcomes of patients treated with the BOLD catheter for symptomatic proximal lower extremity DVT at a single center.

## 2. Materials and methods

### 2.1. Ethics

This retrospective chart review study received approval from the Institutional Review Board. A waiver of informed consent and HIPAA authorization were issued. Formal research ethics committee review, informed consent, or consent for publication were not required. Research was performed according to the principles of the Declaration of Helsinki. All procedures were completed in accordance with local and institutional guidelines.

### 2.2. Study design and population

This study is a retrospective analysis of all consecutive patients treated with PMT using the BOLD catheter by a single interventionalist at one institution between 23 November 2021 and 26 June 2022. At the study institution, patients are selected for interventional DVT treatment in cases of more severe symptomatic presentation. The vascular surgeon in this study decided to treat patients with PMT based on clinical presentation and ultrasound and/or computed tomography venography confirmation of occlusive iliofemoral DVT. Treated patients were those with symptomatic proximal lower extremity DVT involving the common iliac vein (CIV), external iliac vein (EIV), and/or common femoral vein (CFV), with or without involvement of the more distal veins. During the initial experience, severe occlusion of iliofemoral venous segments likely contributed to the decision to employ the BOLD catheter versus other interventional device options used at the study institution, such as the legacy ClotTriever catheter. In addition, the BOLD catheter was generally selected for cases with suspected chronic or mixed chronicity thrombus.

### 2.3. Study outcomes

This analysis aimed to assess the preliminary outcomes using the BOLD catheter as an alternative to the standard catheter in thrombectomy procedures. The primary outcome, intraprocedural safety, was assessed by a chart review of any recorded intraprocedural adverse events or device malfunctions. The secondary outcome was a procedural success, defined as a ≥75% reduction in the total percent venous occlusion across treated venous segments. For each patient, treated venous segments included the CIV, EIV, CFV, and/or FV affected by DVT. When intervening endovascularly for iliofemoral DVT, it is the typical practice of the surgeon to also treat the FV if affected. Procedural success was investigator-assessed through reviewing and comparing pre- and postprocedural venograms. The prethrombectomy total percent venous occlusion of the patients was calculated by averaging the estimated percent occlusion of their treated segments. Procedural success was also examined by the venous segment type.

Additional safety outcomes included complications of the access site through discharge and adverse events at discharge and scheduled 30-day follow-up visits. Adverse events assessed via a chart review included reported acute kidney injury, bleeding events, PE, recurrent DVT, and all-cause mortality. Valve and/or vessel damage was assessed based on venograms. Further outcomes included the ability to complete the procedure in a single session, the length of postprocedural hospital and intensive care unit (ICU) stay, and the reduction of DVT-associated findings on physical examinations during the follow-up visit. Chart notes regarding the physical characteristics of extracted thrombus upon post-thrombectomy visual inspection were also reviewed. Similar to the method used in the ClotTriever Outcomes (CLOUT) registry, a thrombus that was soft, dark red, and jelly-like in appearance was considered acute; a light red thrombus was considered subacute; and a firm, pale, and fibrous thrombus was considered chronic (18). During the preparation of the manuscript, summarized case descriptions for selected patients and available information regarding total follow-up and/or readmissions for all cases were added.

### 2.4. Statistical analysis

Statistical analysis was descriptive. Baseline characteristics, procedural characteristics, and outcomes were summarized as median [interquartile range (IQR)] values or number of observations with the proportion of the total. Data were analyzed using Microsoft Excel for Microsoft 365 MSO (version 2206).

## 3. Results

### 3.1. Patient characteristics

A total of 11 patient cases were included in this retrospective study. Table 1 presents the patient characteristics at baseline. The patients were most commonly referred to vascular surgery from the emergency department (*n* = 5). The median patient age was 65 years, and most patients (63.6%) were women. Three patients had a prior history of DVT, and two patients had concomitant PE at baseline. At baseline, six patients (54.4%) presented with DVT symptoms lasting more than 2 weeks. Prior to the intervention, most patients (81.8%) had already received conservative therapy for their current DVT. All patients received unilateral PMT treatment; nine involved the left lower extremity, and two involved the right lower extremity.

**Table 1:** Baseline characteristics.

Characteristic	Median (IQR) or *n* (%)	*N*
Male sex	4 (36.4)	11
Age (years)	65.0 (59.0–81.0)	11
Prior history of DVT	3 (27.3)	11
Contraindication to thrombolytics	0 (0.0)	11
Prior treatment of current DVT	9 (81.8)	11
Concomitant PE	2 (18.2)	11
Symptom duration (weeks)		11
≤2	5 (45.5)	
>2	6 (54.5)	
Unilateral treatment with PMT	11 (100.0)	11
Provoked DVT	8 (72.7)	11
Anticoagulation regimen at baseline		11
Yes	10 (90.9)	
No	1 (9.1)	
Referred from		11
Emergency department	5 (45.5)	
Hospital inpatient	4 (36.4)	
Transferred from another facility	1 (9.1)	
Wound care	1 (9.1)	
Referred by		11
Hospitalist	5 (45.5)	
Emergency room physician	4 (36.4)	
Podiatry	1 (9.1)	
Vascular surgery	1 (9.1)	

### 3.2. Procedural characteristics and outcomes

#### 3.2.1. Procedural characteristics

Table 2 reports the procedural characteristics. A single interventionalist conducted all procedures. A median of four device passes was performed, with a median thrombectomy time of 30 min. No adjunctive thrombolytics were used, and no patient required a blood transfusion. The median blood loss per procedure was 50 ml. Venoplasty was performed in all procedures, and four patients (36.4%) received stents. In the four cases that involved stenting following PMT and venoplasty, intravascular ultrasound was used to evaluate the extent of underlying compression or residual stenosis warranting a stent and to guide its placement.

**Table 2:** Procedural characteristics and outcomes.

Characteristic	Median (IQR) or *n* (%)	*N*
Completed inpatient	10 (90.9)	11
Single session procedure	11 (100.0)	11
Thrombectomy time (min)	30.0 (20.0–45.0)	11
Procedure time (min)	65.0 (50.0–100.0)	11
Number of device passes	4.0 (4.0–6.0)	11
Blood loss (ml)	50.0 (50.0–100.0)	11
Stent(s) placed	4 (36.4)	11
Venoplasty completed	11 (100.0)	11
Adjunctive thrombolytics used	0 (0.0)	11
Oldest thrombus chronicity on visual assessment		11
Acute	1 (9.1)	
Subacute	0 (0.0)	
Chronic	10 (90.9)	
Postprocedure hospital length of stay (days)	1.0 (1.0–2.0)	11
Postprocedure ICU length of stay (days)	0.0 (0.0–0.0)	11

#### 3.2.2. Intraprocedural safety and success

All 11 thrombectomy procedures were completed without intraprocedural adverse events. No malfunctions or complications related to the use of the device were recorded.

**Figure 1:**
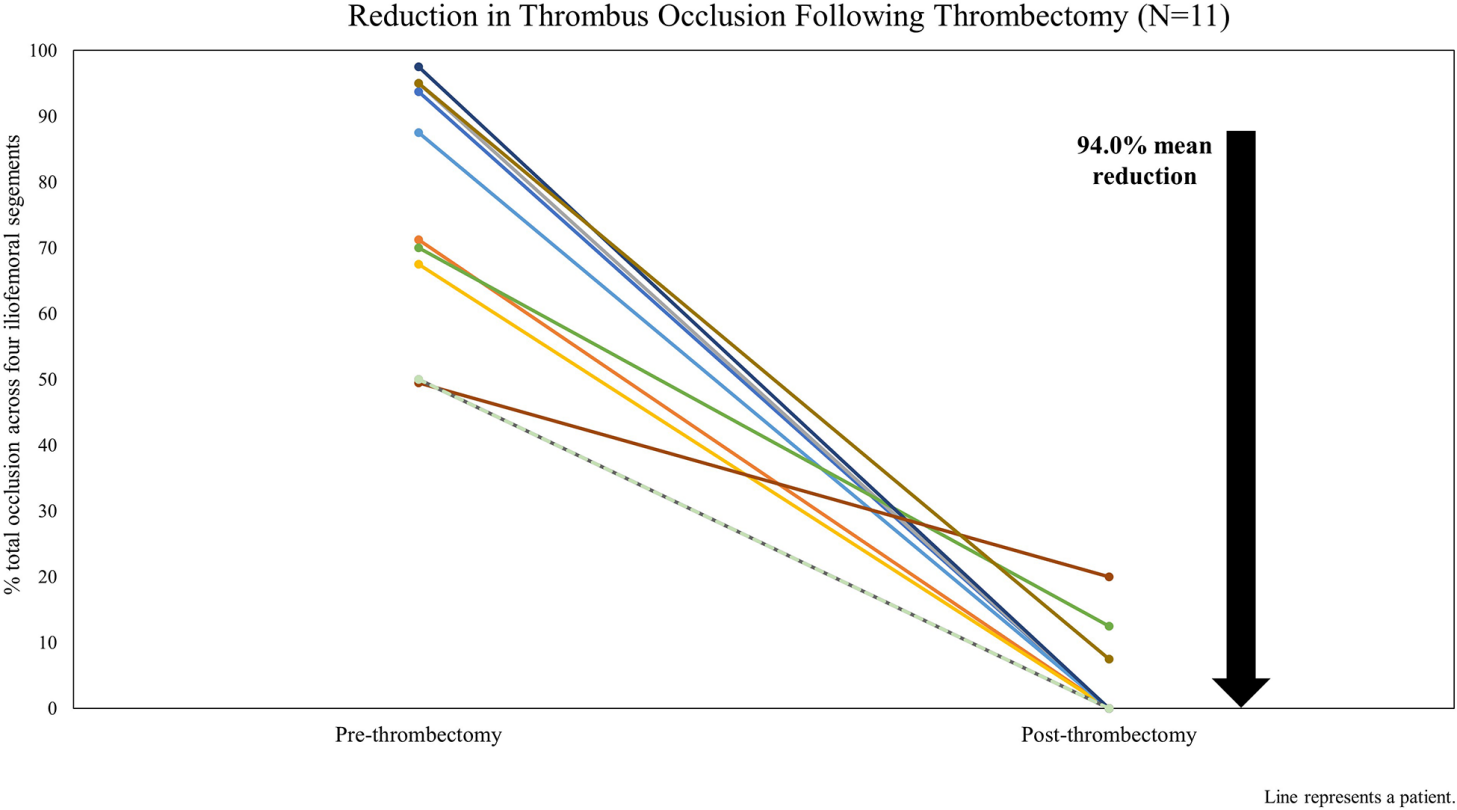
Reduction in iliofemoral thrombus occlusion on procedural venogram following thrombectomy by patient case.

Figure 1 shows the estimated reduction in total thrombus occlusion across iliofemoral venous segments (CIV, EIV, CFV, and FV) pre- and post-thrombectomy based on the venogram review. Procedural success was achieved in 10 cases (90.9%). For Patient 8, the PMT procedure was completed as intended with satisfactory results; however, the resulting occlusion reduction (59.6%) did not meet the definition of procedural success. This patient had preprocedural chronic DVT changes with multiple channels reaching the FV and had residual femoral stenosis following the procedure.

All patients had DVT affecting at least two segments, and five (45.5%) had DVT affecting all four segments. A total of 35 iliofemoral venous segments were treated, and complete resolution of occlusion was achieved in 31 (88.6%). Using the segment type, the median preprocedural vessel occlusion ranged from 90% to 100%, with 100% occlusion of the CFV (*n* = 9) and CIV (*n* = 7). Following the procedure, the median occlusion rate across the four iliofemoral segment types was 0%.

#### 3.2.3. Visual thrombus assessment

Chart notes regarding visual inspection of the extracted thrombus following the thrombectomy were available in all cases. The interventionalist documented that the extracted thrombus contained a chronic component for 10 cases (90.9%) (Table 2): four with acute-on-chronic and six with chronic thrombus. The patient-reported symptom duration at baseline was compared with the recorded chronicity. Chronicity was determined by the observed thrombus consistency after extraction and the presence of collaterals on the venogram. Among the five patients with a baseline symptom duration ≤2 weeks, one patient had an acute thrombus, and four patients (80%) had an extracted thrombus with observed chronic components (two with acute-on-chronic and two with chronic). The cases for the four patients with symptoms persisting for ≤2 weeks and chronic thrombus components (Patients 4, 5, 6, and 7) were presented in greater detail in the following section.

#### 3.2.4. Periprocedural outcomes

All procedures were completed in a single session (Table 2). The median hospital length of postprocedural stay was 1 day, which corresponded to a mean of 1.73 days [standard deviation (SD): ±1.49]. No patients were admitted to the ICU following the procedure. Ten patients were discharged on apixaban, and one was discharged on rivaroxaban. For iliofemoral DVT patients, anticoagulation is typically prescribed for 12 months, and follow-up appointments are scheduled at 1 month and 6–12 months.

### 3.3. Postprocedural outcomes

#### 3.3.1. Safety outcomes

Access site complications were assessed at discharge, and there were no complications among the 10 patients with chart notes specific to access site evaluation. At discharge, no adverse events among the 11 patients included in this study were noted.

Three patients did not complete a follow-up office visit, so their outcomes were limited to those at discharge. Among the eight patients who attended their follow-up visits, there was one adverse event, a recurrent DVT that occurred 13 days postprocedure in a patient with advanced-stage cancer and medication failure. The medication of the patient was changed from apixaban to therapeutic enoxaparin, and additional interventional treatment was not provided. This event was not considered related to the device or procedure. No incidence of vessel damage, valve damage, or acute kidney injury was reported.

#### 3.3.2. Physical examination findings

**Figure 2:**
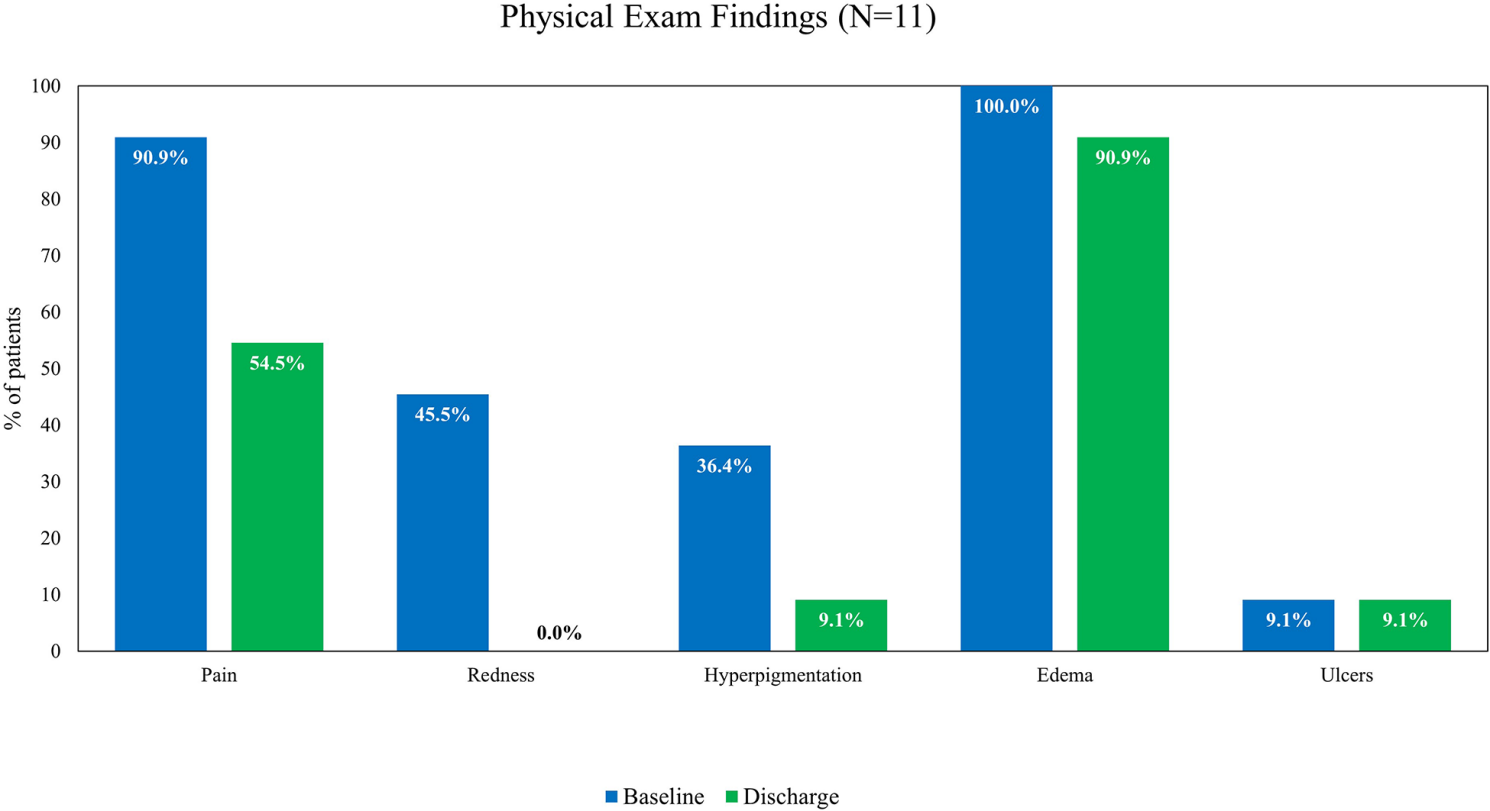
Findings from patient physical examinations from baseline to discharge.

**Figure 3:**
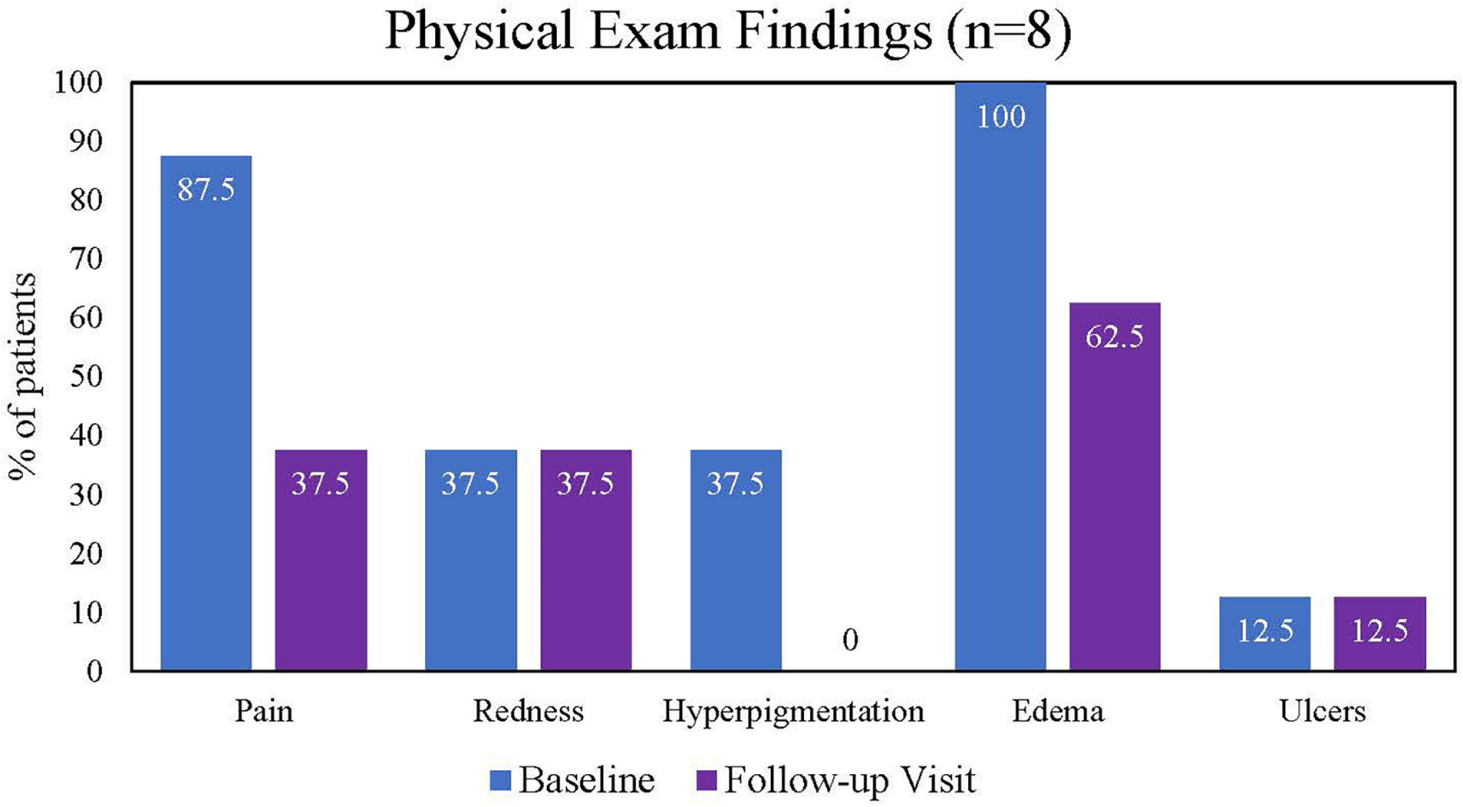
Findings from patient physical examinations from baseline to follow-up visit.

The following physical examination findings recorded at baseline, discharge, and follow-up visits were assessed: pain, redness, hyperpigmentation, edema, and ulcers. At baseline, all patients had edema on physical examination, 10 (90.9%) patients reported pain, and 1 patient had an ulcer. Other frequent findings at the baseline examination included redness (*n* = 5) and hyperpigmentation (*n* = 4). The proportion of patients reporting pain decreased from baseline to discharge (Figure 2) and baseline to the 30-day follow-up visit (Figure 3). All hyperpigmentation had resolved in the three patients with this symptom at baseline, and three patients reported the resolution of their edema prior to the follow-up visit.

### 3.4. Patient cases and follow-up

#### 3.4.1. Case descriptions

The cases for Patients 4, 5, 6, and 7 were selected for summary description because these patients reported symptoms lasting ≤2 weeks, while chart notes regarding visual inspection of the extracted thrombus following thrombectomy indicated a chronic composition.

**Figure 4:**
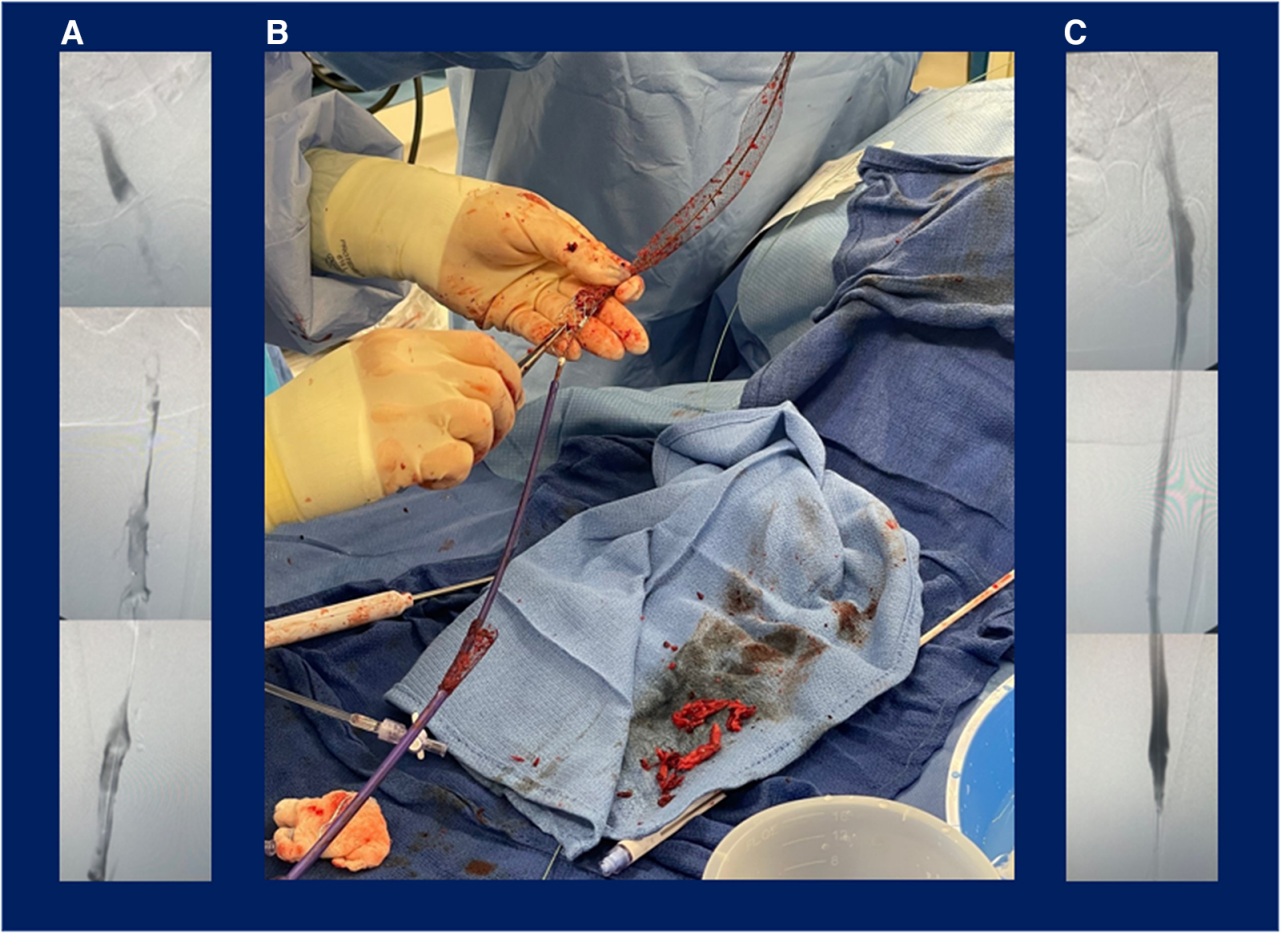
Patient case example: percutaneous mechanical thrombectomy with the ClotTriever BOLD catheter for treatment of deep vein thrombosis. Images from the patient case depict (**A**) preprocedural venogram of extensive deep vein thrombosis, (**B**) procedural use of the ClotTriever BOLD catheter percutaneous mechanical thrombectomy device, and (**C**) postprocedural venogram confirming complete thrombus removal in the treated vessel segments.

Patient 4 was referred from the emergency department, reported acute left lower extremity pain and edema persisting for 1 week, and had not received prior DVT treatment. DVT diagnosis was confirmed via duplex ultrasound and computed tomography scanning. A minor PE was incidentally identified via a computed tomography scan prior to the procedure, and the patient was on a heparin drip at baseline. Six passes with the BOLD catheter were performed during the 65-min procedure, of which the thrombectomy time comprised 35 min. Patient 4 had a 100% reduction of the occlusion across three iliofemoral segments, and the extracted thrombus appeared chronic. Figure 4 shows the intraprocedural images from this case, including the preprocedural venogram (Figure 4A), device image during use (Figure 4B), and postprocedural venogram (Figure 4C). The patient reported a resolution of pain and was discharged on apixaban 1 day postprocedure. This patient missed the scheduled follow-up visit, and no further record was available.

Patient 5 was a hospital inpatient with concomitant PE who experienced right lower extremity edema, pain, heaviness, discoloration, and difficulty ambulating for 2 weeks. This patient had previously received DVT treatment with oral anticoagulation and was on a heparin drip at baseline. Patient 5 had a fully occluded CFV and had 100% occlusion reduction across four iliofemoral segments with a chronic extracted thrombus following the procedure. Except for edema, other symptoms resolved at discharge, which occurred 2 days postprocedure, with apixaban prescription. This patient missed the scheduled follow-up visit, and no further record was available.

Patient 6 presented to the emergency department reporting acute pain, edema, and redness of the left lower extremity persisting for 1 week and had received anticoagulation treatment for their DVT. The patient was on a heparin drip at baseline. Following the procedure, patient 6 had 82.1% occlusion reduction across three iliofemoral segments, and an acute-on-chronic thrombus was extracted. The patient was discharged on apixaban 1 day postprocedure and completed the initial follow-up visit. The last follow-up for this patient was completed 131 days postprocedure, and a duplex ultrasound scan conducted at this visit confirmed vessel patency. No adverse events were recorded during the follow-up.

Patient 7 was referred from the emergency department; reported 1 week of acute left lower extremity pain, edema, and difficulty walking; had received DVT treatment with anticoagulation; and was on a heparin drip at baseline. Patient 7 had full occlusion of the FV, CFV, and CIV. Following thrombectomy, 100% occlusion reduction was achieved across the four treated iliofemoral segments. The patient was discharged on apixaban 1 day postprocedure and did not complete the follow-up visit. The patient was readmitted to the hospital 100 days postprocedure for recurrent DVT and reported compliance with the anticoagulation regimen. Upon case review, the probability of the patient having a hypercoagulable condition or May–Thurner syndrome not identified during the index procedure was considered.

#### 3.4.2. Patient follow-up

Coincidently, three of the four patients whose cases were described above were the patients who missed the follow-up visit (Patients 4, 5, and 7). Patient 7 missed the follow-up visit and was readmitted for recurrent DVT 4 months later. Patients 4 and 5 had no further chart record of related complications or readmission. Subsequently, the follow-up duration was examined for the eight patients who completed their initial follow-up visit (Patients 1, 2, 3, 6, 8, 9, 10, and 11). The median patient follow-up (*n* = 8) was 137 days (IQR: 65.8–247.8), which corresponded to a mean of 156 days (SD: ±113.2). During the available follow-up (*n* = 8), no longer-term treatment-related complications were identified via a chart review. Three patients had readmissions during this period: Patient 1 for an elective procedure at 151 days post-thrombectomy, Patient 10 for an unrelated wound infection at 123 days post-thrombectomy, and Patient 11 for a medical surgery at 143 days post-thrombectomy.

## 4. Discussion

Although many studies have evaluated PMT with the ClotTriever System, this is the first study to report outcomes for a cohort of patients treated with the BOLD catheter. In the present study, no preliminary safety concerns were identified. All reviewed procedures were completed as intended without intraprocedural adverse events or device malfunction and minimal blood loss. The CLOUT registry of 500 patients treated with the legacy ClotTriever catheter reported a serious adverse event rate of 2.6% at discharge and 8.6% at 30 days (12, 13). In this study, no access site complications or adverse events occurred at discharge. Similar to CLOUT, there was no indication of damage to the vessels or valves on the chart and venogram review. During the 30-day follow-up, one patient with advanced-stage cancer experienced a recurrent DVT at 13 days postprocedure, which was not considered to be related to the device or the procedure.

A greater than 75% reduction in the total percent venous occlusion across the treated segments was achieved in 90.9% of BOLD catheter cases included in this analysis. Another single-center retrospective cohort study of 96 acute iliofemoral DVT patients treated with the legacy ClotTriever catheter reported a ≥75% reduction in the thrombus volume, as assessed by the Marder score in 97% of cases (14). In CLOUT, ≥75% reduction in thrombus burden using the Marder score was reported in 91% of treated limbs (12).

Current treatment guidelines recommend anticoagulation treatment for patients with acute DVT, as evidence supporting the routine use of interventional therapy in typical DVT cases is limited (19, 20). Patients in this study were selected for treatment with the study device based on clinical presentation and ultrasound and/or computed tomography venography confirmation of occlusive DVT affecting the iliofemoral venous segments. This study was exemplified by the median vessel occlusion observed on preprocedural venograms, which ranged from 90% to 100% across the CIV, EIV, and CFV. In this study, most patients (81.8%) received prior anticoagulation treatment for their current DVT, and six (54.5%) reported DVT symptoms that lasted >2 weeks. Thrombi that form in the deep veins undergo relatively rapid compositional changes as fibrin is replaced by collagen (21). Approximately 2 weeks after forming, the majority of a DVT thrombus is composed of collagen, with a collagen composition as high as 80% by week 3 (22). This change in composition leads to a thrombus unable to be resolved by natural processes, even in the presence of anticoagulants, or by treatment with fibrinolytics.

Before introducing the BOLD catheter, the treating surgeon noted that an iliofemoral DVT thrombus extracted by PMT using the ClotTriever System commonly had some chronic components, even among patients with an acute symptom onset. This anecdotal observation was confirmed by recent results from the CLOUT registry and *post-hoc* analysis of the CAVA study, showing that a chronic DVT thrombus is more common than expected based on the symptom duration alone (11, 23). The results of this study similarly support this finding as a thrombus with chronic elements was extracted in most cases (90%), including four out of the five patients with a DVT symptom duration ≤2 weeks. The frequent extraction of a chronic iliofemoral DVT thrombus contributed to the rationale for initiating this retrospective case review because there are few endovascular treatment options for patients with chronic DVT, which greatly reduces the quality of life and increases morbidity risks (24, 25). The preliminary procedural safety and success outcomes provided by this study justify the further collection of safety and effectiveness data using the BOLD catheter in larger patient populations.

The purpose of this analysis was limited to assessing the preliminary outcomes of patients treated using the BOLD catheter. This study has several limitations, including the retrospective nature of the analysis, which was conducted in a small population of patients treated at one center by a single interventionalist without a control group. In addition, not all patients completed the scheduled follow-up visits. Among those who did, the follow-up duration was short at 30 days. In this study, most patients received prior anticoagulation therapy for their current DVT, and all were symptomatic with occlusive iliofemoral DVT confirmed via imaging. However, it is well recognized that there is an overall need for further studies of PMT versus alternative DVT treatment options, and no conclusions of comparative safety or effectiveness can be drawn from this study. Evaluating the extracted DVT thrombus material has only recently been possible using PMT since previous studies of interventional DVT treatment used catheter-directed thrombolysis. Although treating non-acute DVT is of interest due to the limited availability of therapeutic options for these patients, no method for assessing the chronicity of the extracted DVT thrombus has been validated in the literature.

## 5. Conclusion

The preliminary data from initial experience with the ClotTriever BOLD catheter at the study institution indicate successful PMT completion in a small series of patients with symptomatic proximal lower extremity DVT. There were no intraprocedural adverse events, and a ≥75% reduction in occlusion across iliofemoral venous segments was achieved in 10 (90.9%) cases. No postprocedural safety concerns were identified via a chart review. These results justify the further collection of safety and effectiveness study data in a larger patient population.

## Data Availability

The raw data supporting the conclusions of this article will be made available by the authors, without undue reservation.
